# Therapeutic Strategies for Spinal Cord Injury

**DOI:** 10.3390/ijms19103200

**Published:** 2018-10-16

**Authors:** Pavla Jendelova

**Affiliations:** 1 Institute of Experimental Medicine, Czech Academy of Sciences, Vídeňská 1083, 142 20 Prague, Czech Republic; pavla.jendelova@iem.cas.cz; Tel.: +420-241-062-828; Fax: +420-241-062-782; 2 Department of Neuroscience, 2nd Faculty of Medicine, Charles University, V Úvalu 84, 150 06 Prague, Czech Republic

The first description of spinal cord injury (SCI) comes from the Edwin Smith papyrus, dating from seventeenth century “B.C”. In this unique treatise, SCI was considered as an ailment that could not be treated [1]. To date, we still do not have the tools needed to regenerate nervous tissue. However, different approaches and strategies continue to emerge, putting pieces of knowledge together and trying to challenge SCI and improve patients’ quality of life. Modern techniques have been employed to analyze the changes after SCI. Some of the new approaches and strategies are described or summarized in this Special Issue. The combination of transcriptomics, proteomics, and bioinformatics provides a comprehensive overview of proteins with persistent differential expression at the mRNA and protein level, and from the subacute (7 days) to the chronic (8 weeks) phase of SCI lesion development. A combined analysis identified 40 significantly upregulated versus 48 significantly downregulated molecules. This screening revealed several possible therapeutic candidates, which were so far not considered as potential targets, but they possess important bioactivity, such as the upregulated purine nucleoside phosphorylase (PNP), cathepsins A, H, Z (CTSA, CTSH, CTSZ), and proteasome protease PSMB10, as well as the downregulated ATP citrate lyase (ACLY), malic enzyme (ME1), and sodium-potassium ATPase (ATP1A3) [2].

The first assessment of pro- and anti-inflammatory cytokines, depending on the site of injury, revealed differences in Vascular Endothelial Growth Factor (VEGF), leptin, Interferon gamma-induced protein 10 (IP10), Interleukin 8 (IL18), Granulocyte-colony stimulating factor (GCSF), and fractalkine in thoracic and cervical lesions. Overall, cervical SCI had reduced expression of both pro- and anti-inflammatory proteins relative to thoracic SCI [3]. In response to the release of inflammatory cytokines, stress-activated protein kinases (SAPKs)—c-Jun N-terminal kinase (JNK) and p38 mitogen-activated protein kinase (p38 MAPK)—are activated in various types of cells. In animal models of SCI, the inhibition of either JNK or p38 has been shown to promote neuroprotection-associated functional recovery. Therefore, p38 could serve as a promising target for therapeutic intervention in SCI [4]. Among the drugs used for other pathologies, atorvastatin (ATR)—a potent inhibitor of cholesterol biosynthesis—can modulate secondary injury, reducing Interleukin 1 (IL1), M1 macrophage infiltration, and decreasing the activity of caspase 3. These changes lead to increased sprouting and improved locomotor activity [5]. Pharmacological treatment can be combined with stem cells, but these therapies do not always have to synergize, as in the case of ependymal stem/progenitor cells combined with a pharmacological compound FM19G11, which reduced glial scar and increased the expression of Olig1 in vivo, but did not lead to greater behavioral improvement when compared with the individual treatments [6]. Stem cells have promising therapeutic potential to rescue or repair damaged spinal cord tissue. Particularly, endogenous stem cell populations have been considered as a promising therapeutic approach to enhance repair mechanisms in SCI. Their potential is reviewed in [7]. However, further investigations are necessary to confirm the neurological benefits by adjusting the doses and time points for the administrations of stem cells. It has been confirmed that the efficacy of cell therapy is dose-dependent and can be enhanced by repeated applications, as was shown in a study of Wharton Jelly mesenchymal stem cells (WJMSCs) grafted into acute spinal cord balloon compression lesions. Histochemical analyses revealed a gradually increasing effect of grafted cells, resulting in a significant increase in the number of sprouting fibers, a higher amount of spared gray matter, and reduced astrogliosis. mRNA expression of macrophage markers and apoptosis was downregulated after the repeated application of 1.5 million cells [8]. An attractive option as an alternative to cell therapy is the use of stem cell secretomes, since the immunomodulatory and neurotrophic properties of stem cells rely on released secretomes, comprising a soluble fraction of proteins, growth factors, cytokines, miRNA, and other bioactive molecules. Indeed, an effect similar to cell grafting was obtained after the repeated intrathecal delivery of conditioned media obtained from bone marrow mesenchymal stem cells [9]. It is important to highlight that stem cell-based therapy alone is not sufficient to bridge a spinal cord lesion. Therefore, a repair strategy based on a combination of well-established therapeutic modalities, including surgery and medications, and/or bridging the lesion with biocompatible scaffolds, is another approach for the treatment of SCI. Methacrylate hydrogels are biocompatible polymers used for bridging large cavities. They can be coated or modified with extracellular matrix (ECM) components such as laminin and fibronectin, which can improve cell adhesion and survival by generating a permissive microenvironment within the biomaterial. Hydrogels based on hydroxypropylmethacrylamid (HPMA) and 2-hydroxyethylmethacrylate (HEMA) coated with fibronectin support the ingrowth of axons and blood vessels when grafted into rat hemisection [10].

Perineuronal nets (PNNs) are extracellular matrix structures surrounding neuronal sub-populations throughout the central nervous system, regulating plasticity. Enzymatically removing PNNs successfully enhances plasticity and thus functional recovery, particularly in models of spinal cord injury. While PNNs within various brain regions are well-studied, much of the composition and associated populations in the spinal cord is as yet unknown. Kwok’s lab investigated the populations of PNN neurons involved in functional motor recovery. Insights into the role of PNNs and their molecular heterogeneity in the spinal motor pools could aid in designing targeted strategies to enhance functional recovery post-injury [11].

Neuropathic pain after spinal surgery—the so-called failed back surgery syndrome—is a frequently observed common complication. One cause of the pain is scar tissue formation, observed as post-surgical epidural adhesions. These adhesions may compress the surrounding spinal nerves, resulting in pain, even after successful spinal surgery. An anti-adhesive membrane can reduce adhesions and scar formation and lower the numbers of fibroblasts and inflammatory cells [12]. Neuromuscular impairment and reduced musculoskeletal integrity are hallmarks of SCI that hinder locomotor recovery. Activity-based physical rehabilitation therapies (ABTs) can promote neuromuscular plasticity after SCI. However, ABT efficacy declines as SCI severity increases. Additionally, many men with SCI exhibit low testosterone, which may exacerbate neuromusculoskeletal impairment. Incorporating testosterone adjuvant to ABTs may improve musculoskeletal recovery and neuroplasticity, as androgens attenuate muscle loss and promote motoneuron survival. In a review [13], the mechanisms and benefits of a multimodal strategy involving ABT with adjuvant testosterone are discussed.

Despite all the promising results in preclinical research, translation into the clinic is progressing slowly. The most promising clinical trials and biomaterials with high translational potential are presented in a review [14]. To date, the majority of patients remain in a chronic state for the rest of their lives. To solve this so-far incurable condition, we need to include more clinically relevant chronic SCI models, which would allow a reliable assessment of the therapeutic strategies for future treatments of SCI patients.
